# The Roles of MicroRNAs in Tendon Healing and Regeneration

**DOI:** 10.3389/fcell.2021.687117

**Published:** 2021-07-02

**Authors:** Lingli Ding, Min Wang, Shengnan Qin, Liangliang Xu

**Affiliations:** ^1^Lingnan Medical Research Center, The First Affiliated Hospital, Guangzhou University of Chinese Medicine, Guangzhou, China; ^2^Department of Orthopaedics, Guangzhou Institute of Traumatic Surgery, Guangzhou Red Cross Hospital, Medical College, Jinan University, Guangzhou, China

**Keywords:** microRNA, tendon healing, tendinopathy, tenogenesis, stem cells

## Abstract

Tendons connect the muscle abdomen of skeletal muscles to the bone, which transmits the force generated by the muscle abdomen contraction and pulls the bone into motion. Tendon injury is a common clinical condition occurring in certain populations, such as repeated tendon strains in athletes. And it can lead to substantial pain and loss of motor function, in severe cases, significant disability. Tendon healing and regeneration have attracted growing interests. Some treatments including growth factors, stem cell therapies and rehabilitation programs have been tried to improve tendon healing. However, the basic cellular biology and pathology of tendons are still not fully understood, and the management of tendon injury remains a considerable challenge. Regulating gene expression at post-transcriptional level, microRNA (miRNA) has been increasingly recognized as essential regulators in the biological processes of tendon healing and regeneration. A wide range of miRNAs in tendon injury have been shown to play vital roles in maintaining and regulating its physiological function, as well as regulating the tenogenic differentiation potential of stem cells. In this review, we show the summary of the latest information on the role of miRNAs in tendon healing and regeneration, and also discuss potentials for miRNA-directed diagnosis and therapy in tendon injuries and tendinopathy, which may provide new theoretical foundation for tenogenesis and tendon healing.

## Introduction

Tendon is a connective tissue composed of closely arranged bundles of parallel collagen fibers (Wang et al., 2012). It plays an important role in the skeletal muscle system. Tendons are located between muscles and bones and can cushion the pressure caused by the direct interaction between muscles and bones, thereby stabilizing the joints (Laurencin and Freeman, 2005; Thorpe and Screen, 2016). As a long-term pressure-bearing tissue, tendons are easy to form a variety of acute and chronic injuries (Khan et al., 1999). Since the beginning of this century, the global prevalence of tendon disease has been on the rise. Tendon disease is one of the most common diagnoses of people engaged in sports professions, accounting for 30% of the total number of injuries diagnosed (Millar et al., 2021). At the late stage of tendon healing, it is easy to form scar tissue with decreased strength. And it is easy to adhere to the surrounding tissue, which increases the risk of tendon injury again (Bruns et al., 2000; Sharma and Maffulli, 2005). On the other hand, there are fewer blood vessels and poor blood flow inside the tendon, which makes the tendon heal more slowly (Whalen, 1951; Miyashita et al., 1997; Tempfer and Traweger, 2015; Nichols et al., 2019). The high frequency and slow healing of tendon injury not only seriously affects the normal life of patients, but also increases the social and economic burden. Therefore, how to promote tendon repair and regeneration is a great challenge in tissue engineering. miRNA is a small non-coding ribonucleic acid that participates in the regulation of cellular processes by inhibiting the translation and stability of messenger ribonucleic acid. Since the discovery of miRNA in the 1990s, people’s understanding of miRNAs has been deepened (Lee et al., 1993; Di Leva et al., 2014), and its application has become more and more extensive. miRNAs play an important role in inflammation, cell cycle regulation, stress response, cell growth, differentiation, proliferation, apoptosis and migration etc. (Lee et al., 1993; Hata, 2013; Saliminejad et al., 2019), recent studies have shown that they also participate in tendon regeneration and repair.

Here, we briefly described the structure and function of tendon, and the biology of miRNA. Then the roles of miRNA in tendon repair and regeneration was summarized; and the potentials and challenges of miRNA-directed diagnosis and therapy in tendon injuries and tendinopathy were also discussed.

## Tendon Structure

Tendon is a connective tissue rich in matrix, which is mainly composed of closely arranged bundles of parallel collagen fibers (Wang et al., 2012; Tsai et al., 2021). They are attached to muscles and bones, and stabilize joints by cushioning the pressure caused by direct interaction between muscles and bones (Laurencin and Freeman, 2005; Thorpe and Screen, 2016). The place where the tendon is inserted into the muscle is called the tendon joint, while the joint between the tendon and the bone is called the bone-tendon junction (O’Brien, 1997; Thorpe and Screen, 2016). The tendon, as a tissue that transmits and loads, transfers strength from the muscle to the bone and drives the joint movement by contracting the muscle (Wang, 2006; Voleti et al., 2012). There are usually no blood vessels inside the tendon, while there are blood vessels and nerves passing through the sheath around it (Kannus, 2000; Tempfer and Traweger, 2015).

Tendon is mainly composed of type I collagen (also known as collagen I, COL I), which contains a small amount of proteoglycans, glycoproteins and minor collagens (Voleti et al., 2012). The dry mass of human tendons accounts for about 30% of the total tendon mass, and the remaining 70% is water (Sharma and Maffulli, 2005). Type I collagen accounts for 65–80%, and elastin accounts for about 2% of the dry weight of tendons (Sharma and Maffulli, 2006). Collagen is mainly a helical structure formed by the intertwining of three chains (Voleti et al., 2012). It is constantly cross-linked to form the specific spatial structure of the tendon (Gaut and Duprez, 2016). The collagen fibers are considered to be the basic force transmission units of tendons, which are densely arranged in the extracellular matrix (ECM) and parallel to the bone-muscle axis (Nourissat et al., 2015). Among the tendon cells, tenoblasts and tenocytes accounts for about 90%, while the remaining 10% are mainly composed of chondrocytes, synovial cells, vascular endothelial cells and smooth muscle cells (Sharma and Maffulli, 2006; Wang, 2006).

### MicroRNA Biology

Since the discovery of the first miRNA in 1993 and another miRNA let-7 7 years later (Lee et al., 1993; Reinhart et al., 2000; Di Leva et al., 2014), people’s understanding of miRNA has been deepened for more than two decades, and the potential therapeutic effects of miRNA have been explored. According to the latest version of the microribonucleic acid database (miRBase) released in 2018, the human genome contains 1,917 annotated hairpin precursors and 2,654 mature sequences (Kozomara et al., 2019). miRNA is a class of endogenous non-coding RNA molecules with a length of about 22 nucleotides (Ambros et al., 2003; Ambros, 2004; Kabekkodu et al., 2018; Lu and Rothenberg, 2018). miRNA regulates gene expression mainly by binding to mRNA, which plays an important role in cell growth, differentiation, proliferation and apoptosis (Vasudevan et al., 2007; Sayed and Abdellatif, 2011). They are widely distributed in many tissues in the body which can be extracted from cells, tissues and body fluids (tears, urine, plasma and serum) (Lu and Rothenberg, 2018). Most of the miRNAs are transcribed by the ribonucleic acid polymerase II (Cai et al., 2004; Lee et al., 2004), but there is a small part of miRNAs that are associated with Alu repeats have been reported to be transcribed by Pol III (Borchert et al., 2006). The production of miRNA is initiated by the transcription of pri-miRNA in the nucleus. Before being transported to the cytoplasm, pri-miRNA is further processed by RNase III Drosha enzyme to form miRNA precursor (pre-miRNA). Then it is digested by another RNAse III enzyme Dicer, and binds with Argonaute protein to form RNA-induced silencing complex (RISC). After that, one of the double strands is degraded and the other forms a mature miRNA (Esteller, 2011; Di Leva et al., 2014; Koturbash et al., 2015; Vidigal and Ventura, 2015). Although the miRNAs do not encode proteins themselves, they could influence the transcription of mRNAs to regulate proteins expression (Selbach et al., 2008). The functions of miRNA are mainly divided into four aspects: (1) participate in epigenetic regulation of gene expression; (2) mRNA degradation; (3) post-transcriptional inhibition of target mRNA translation; (4) act as a ligand to bind to immune receptors and participate in inflammatory response (Carthew and Sontheimer, 2009; Fabbri et al., 2012; Lehmann et al., 2012; Fritz et al., 2016; Kabekkodu et al., 2018). Therefore, exploring new means of treating diseases are made possible by targeting miRNAs to intervene in the activity of specific genes. This strategy is also beneficial for promoting tendon healing and regeneration which is linked to the roles of miRNAs in tendon.

## Micrornas Regulate Tendon Healing

Tendon healing is characterized by scar formation, tissue disorder, and decreased mechanical properties (Andarawis-Puri et al., 2015). Tendon healing can be generally summarized as three stages: inflammatory response stage, proliferative repair stage and remodeling stage, and these three stages overlap each other (Strickland, 2000). Tendinopathy is a failed healing reaction, accompanied by haphazard proliferation of tendinocytes, intracellular abnormalities, destruction of collagen fibers, and subsequent increase in the non-collagen matrix (Longo et al., 2018). Many miRNAs have been shown to be involved in regulating the biological processes of tendon healing (Summarized in Table 1).

**TABLE 1:** Summary of miRNAs in tendon tendinopathy.

**miRNA**	**Targets**	**Models**	**Cells**	**Biological functions**	**References**
miRNA-499	CUGBP2, MYB	Clinical samples of tendinopathy		Regulated cell proliferation, tenocyte apoptosis and differentiation	Cai et al., 2015
miR-205-5p	VEGFA		Rats BMSCs	involved in tendon-bone healing of RCT	Xu et al., 2019
miR-146a-5p, miR-193b-3p, etc.	JAK2/STAT3 and interconnecting pathways	Clinical Human biceps tendons		Linked to inflammation	Thankam et al., 2018
miR-31-5p, miR-195-5p, etc.	AMPK and TREM-1 signaling	Clinical Human rotator cuff tendon injuries		Associated with the pathogenesis of RCT injuries with fatty infiltration and inflammation	Thankam et al., 2019
miR-29b	TGF-β1/Smad3	Achilles tendon injury rats		Inhibition of fibroblasts growth.	Chen et al., 2014
miR-21-5p	Smad7	tendon adhesion in mice	Tenocytes and fibroblasts	Controlling the fibrotic healing response	Cui et al., 2019
miR-337-3p	IRS1 and Nox4		Rat TSPCs	Alleviating ectopic ossification in rat tendinopathy	Geng et al., 2020
miR-21-3p	p65		HUMSC-Exos	tendon adhesion	Yao et al., 2020
miR-145-5p, miR-151a-3p, miR-382-5p, miR-199a-5p, miR-21-5p, miR-125a-5p, and miR-498	COL1A2, COL3A1, MMP9 and MMP2	Clinical human shoulder tendons		Associated with the integrity of tendon matrisome	Thankam et al., 2016
miR-608	COL5A1		HT1080 fibrosarcoma cells	Associated with the molecular basis of tendinopathy	Abrahams et al., 2013
miR-29a	IL-33/sST2	Superficial digital flexor tendon of horses	Human tenocytes	Facilitating tissue remodeling in the tendon after injury	Millar et al., 2015; Watts et al., 2017
miRNA-338, miRNA-381	Scx	Untrained adult rats under a single session of mechanical loading	Tendon fibroblast	Regulating development of limb tendons	Mendias et al., 2012
miR28-5p	p53 deacetylase sirtuin 3		Primary human tenocytes	Prevention of bim RNA Degradation	Poulsen et al., 2014
miR-148a-3p	KLF6		A co-culture system of tenocytes with ECs	Correlated with Tsp-4 levels and promoting angiogenesis	Ge et al., 2018
miR-210	VEGF, FGF2 and COL 1	Transected and repaired rats achilles tendons		*via* acceleration of angiogenesis	Usman et al., 2015

### miRNAs Regulate Cellular Proliferation and Inflammation

Apoptosis of tendinocytes is accelerated within a few days after injury, followed by increased cell proliferation within 2–4 weeks, which activates molecular events to inhibit apoptosis (Wu et al., 2010). One study filtrated differentially expressed genes between diseased and normal tendons, and miR-499 was found to regulate CUGBP2 and MYB which are important regulators of cellular proliferation, apoptosis and differentiation (Cai et al., 2015). Inhibiting miRNA-205-5p could promote tendon-bone healing of rotator cuff tendon (RCT) through increasing cellular viability and proliferation (Xu et al., 2019).

It has been reported that inflammation is involved in the whole process of tissue repair, with both advantages and disadvantages (Eming et al., 2009). The expression levels of miRNAs that are inflammatory targets mediated by the JAK2/STAT3 pathway, such as miR-146a-5p, miR-193b-3p, etc., were significantly reduced in glenohumeral arthritis tendon (Thankam et al., 2018). The network analysis of genes associated with AMPK and TREM-1 signaling revealed miR-31-5p, miR-195-5p and other thirteen miRNAs might be interrelated with the pathogenesis of RCT injury patients (Thankam et al., 2019). Such knowledge has important implications for inflammatory response and proliferative repair stage of tendon healing.

### miRNAs Regulate Tendon Adhesion

Despite advances in tendon repair and post-operative rehabilitation, tendon adhesion is still considered to be a challenging part of the repair process. The formation of adhesion during tendon healing is regulated by TGF-β, Smad3, p65, etc. (Katzel et al., 2011; Wu et al., 2016; Chen et al., 2017). Using miRNA therapy to modulate TGF-β expression holds great promise in preventing tendon adhesion formation (Chen et al., 2009). Overexpression of miR-29b down-regulated TGF-β1/Smad3 levels, and inhibited fibroblast growth, thereby enhanced tendon healing after rats Achilles tendon injury (Chen et al., 2014). Exosomes are rich in miRNAs, which act as important regulators of intercellular communication and play an irreplaceable role in inflammation, fibrogenesis, and tissue repair (Simeoli et al., 2017). Exosomal miR-21-5p secreted by bone marrow macrophages directly targets Smad7 and leads to increased proliferation and migration ability of tenocytes as well as fibrosis activity, providing a potential target for the prevention and treatment of tendon adhesion (Cui et al., 2019). In addition, a study has shown that human Umbilical Cord Stem Cell-Derived Exosomes may regulate p65 activity through the delivery of miR-21a-3p, and ultimately inhibit tendon adhesion (Yao et al., 2020).

These studies are promising for further research into tendon adhesion, and are critical to determine how to improve repair outcomes and identify new therapeutic strategies to promote tendon healing and prevent adhesion formation.

### miRNAs Regulate Tendon Extracellular Matrix

Tenocytes produce ECM which participates in tendon injury repair to maintain tendons homeostasis. The matrix remodeling rate of pathological tendons is increased, which leads to the decrease of mechanical stability of tendons and more vulnerable to injury (Longo et al., 2018). ECM disrupted by matrix metalloproteinases is another mark for ECM remodeling occurring at the site of the lesion (Xue and Jackson, 2015). Seven miRNAs, including miR-145-5p, miR-151a-3p, miR-382-5p, miR-199a-5p, miR-21-5p, miR-125a-5p, and miR-498 were found to be highly active and are thought to mediate major biological processes related to tendon matrix body integrity, which may be associated with tendon-related pathology (Thankam et al., 2016). COL5A1 encodes the α1 chain of type V collagen which is a minor amount fibrillar collagen (Posthumus et al., 2011). In its 3′-untranslated region (3′-UTR), COL5A1 gene has a *Bst*UI restriction fragment length polymorphisms, which is associated with achilles tendon pathology and more specifically, chronic achilles tendinopathy (Mokone et al., 2006). miRNA can inhibit protein synthesis by binding to COL5A1 3′-UTR to regulate target mRNA stability and/or translation efficiency (Garzon et al., 2009). A functional miRNA site for miR-608 within the COL5A1 3′-UTR was identified, which has important implications for the understanding of the molecular basis of tendinopathy and other exercise-related phenotypes (Abrahams et al., 2013). miR-29a demonstrated a functional role in post-transcriptional regulation of collagen in murine and human (Millar et al., 2015).

### miRNAs Regulate Tendon Homeostasis

Some studies have highlighted miRNAs involved in tendon homeostasis during developmental and healing processes. Skeletal muscle has intimate functional adaptations with tendon, and they are called as “tendon units” (Magnusson et al., 2008). miRNAs including miRNA-1, miRNA-133a, miRNA-133a^∗^ and miRNA-133b, which regulate striated muscle to mechanical loading, unloading and various disease processes (McCarthy et al., 2009; McCarthy, 2011), are all increased in mechanically loaded tendons and TGF-β-treated fibroblasts (Mendias et al., 2012). Mechanosensitive miRNAs, including miR-338, and miR-381, may bind to the 3′-UTR of scleraxis (Scx), which is a key regulator of extremity tendon development (Mendias et al., 2012). miR-100, miR-378, miR-205, miR-21, miR-221, and miR-222 were shown to involve with the accommodation of ECM synthesis and cell proliferation. And the let-7 family (such as let-7a, let-7e, and let-7b) also plays significant role in adjusting postnatal tendon adaptation to mechanical loading and TGF-β treatment (Mendias et al., 2012). Overexpression of mechanical sensitive miR-337-3p mitigates ectopic ossification in rat tendinopathy model *via* targeting insulin receptor substrate 1 (IRS1) and NADPH oxidase 4 (Nox4) of tendon derived stem cells, which not only provides a new understanding of the molecular mechanism of heterotopic ossification in tendinopathy, but also emphasizes the significance of miR-337-3p as a recognized therapeutic target for the clinical treatment of tendinopathy (Geng et al., 2020). Tenocytes routinely encounter oxidative stress. High glucose combined with oxidative stress lead to the up-regulation of miR28-5p which directly down-regulates the expression of p53 deacetylase sirtuin 3, leading to the increase of p53 acetylation level (Poulsen et al., 2014).

Thrombospondin-4 (Tsp-4) is indispensable for muscle attachment and ECM assembly at muscle-tendon junctions (Subramanian and Schilling, 2014). Targeting Krüppel-like factor 6 (KLF6), miR-148a-3p could affect the expression of TSP-4 in tendonocytes, and is closely related with Tsp-4 levels in tendinopathy tissues, which also promoted endothelial cell (EC) angiogenesis (Ge et al., 2018). Single local injection of double stranded miR-210 accelerated neovascularization through upregulating the expression of VEGF, FGF2 and type I collagen (COL1), and induced a microenvironment conducive to rat Achilles tendon healing during the early phase (Usman et al., 2015). The formation of new capillaries through angiogenesis is the key to tendon healing. However, excessive or dysfunctional angiogenic responses may adversely affect the healing outcome.

Understanding the relationship between miRNAs and the biological elements of tendon development, tendon homeostasis, and healing is the first requirement to determine an effective treatment for tendinopathy.

## Micrornas in Tenogenic Differentiation

The aim of tendon regeneration is to restore its inherent structural and functional characteristics, which remains a great challenge (Andarawis-Puri et al., 2015). Stem cell-based therapy has become an important research direction in tissue engineering and regenerative medicine, especially for tendon and bone. Mesenchymal stem cells (MSCs) and tendon stem/progenitor cells (TSPCs) have received increasing attention toward tenogenic differentiation and tendon regeneration. MSCs are self-renewing, cultured and extended adult stem cells isolated from a variety of tissues with the ability of multipotent differentiation (Ferreira et al., 2018). TSPCs referred to as tendon-derived stem cells (TDSCs) or tendon stem cells (TSCs). These cells, residing in the fascicular matrix of tendon, also have self-renewal and multi-lineage differentiation ability, and might open a new field of tenogenesis and replacement of damaged tendons (Zhou et al., 2010). Current studies with respect to the effect of miRNAs known to be involved in tenogenesis were summarized in Table 2.

**TABLE 2:** Summary of miRNAs in tenogenic differentiation (+).

**miRNA**	**stem cells**		**Targets**	**Tenogenic differentiation**	**References**
	**Cell type**	**Tissue origin**			
					
miR-140-5p	TSPCs	Human tendons	Pin1	(−)	Chen et al., 2015a
miR-29b-3p	MSCs and TSPCs	Human	TGF-β1 and COL1A1	(−)	Lu et al., 2017
miR-378a	TSPCs	Mice tail tendons	TGF-β2	(−)	Liu et al., 2019
miR124	TSPCs	Human Anterior Cruciate Ligament	EGR1	(−)	Wang et al., 2016
let-7	TSPCs	Human	HMGA2	(−)	Sun et al., 2020
miR-135a	TSPCs	Rat Achilles tendons	ROCK1	(−)	Chen et al., 2015b
miR-217	TSPCs	Human Achilles tendons	p16	(+)	Han et al., 2017
miR-218	BMSCs	Rats	TOB1	(+)	Gao et al., 2016

*(+) and (−) represent promotion and inhibition, respectively.*

### miRNAs Inhibit Tenogenic Differentiation

It is well known that TGF-β1 is produced by fibroblasts and can be upregulated in autosynthesis, and overproduction of TGF-β1 leads to excessive cell proliferation and matrix synthesis (Tomasek et al., 2002; Frangogiannis, 2020). Type I collagen (COL1), forming macromolecular networks, is the most abundant protein in the human body and provides strength and resiliency to tissues such as tendons, and ligaments (Rittié, 2017). miR-29b-3p has been reported to directly inhibit the expression of TGF-β1 and COL1, thereby forming a new regulatory feedback loop between H19 and TGF-β1 and inhibiting tendon differentiation (Lu et al., 2017). Transforming growth factor β2 (TGF-β2) is required for fetal tendon development in mice and had been shown to induce Scx expression in mouse and chick embryos (Pryce et al., 2009; Havis et al., 2016). Embedding in the sequence of this transcriptional regulator of oxidative energy metabolism is miR-378a, which is involved in metabolic pathways, mitochondrial energy homeostasis, and related biological processes such as muscle development, differentiation, and regeneration (Krist et al., 2015). In miR-378a knock-in transgenic mice and their TSPCs, miR-378a impaired tenogenic differentiation and tendon injury healing by inhibition collagen and ECM production *via* reducing TGF-β2 (Liu et al., 2019). Early growth response-1 (EGR-1) regulated specific differentiation of TSPCs into tenocytes and also induced the expression of tendon marker genes SCX, TNMD, TNC, and COL1 both *in vitro* and *in vivo* (Tao et al., 2015). Directly binding with EGR-1, miR-124 prevented collagen formation and tendon differentiation *via* suppressing EGR-1 expression (Wang et al., 2016). The high-mobility AT-hook 2 (HMGA2) mRNA transcript, involved in many cellular processes, contains seven complementary sites for let-7 binding in its 3′ -UTR and is known for its regulatory role in stem cell self-renewal and differentiation along with oncogenesis (Hammond and Sharpless, 2008). Recent studies have established that HMGA2 overexpression protected hTSPCs against H_2_O_2_-induced loss of stemness through autophagy activation, while increased HMGA2 silencing by the miRNA let-7 could induce hTSPC impairments, and thus plays a critical role in tendon homeostasis and regeneration (Sun et al., 2020). Rho-associated coiled-coil protein kinase 1 (ROCK1) plays vital roles in many aspects including cell morphology, mitosis, motility, and even senescence (Julian and Olson, 2014; Guan et al., 2020). miR-135a which directly binded to the 3′-UTR of ROCK1, suppresses proliferation, migration and tenogenic differentiation of TSPCs (Chen et al., 2015b). miR-140-5p has been shown to delay the progression of human TSPCs senescence by targeting Pin1 which is a highly conserved peptidyl-prolyl isomerase (PPI) (Chen et al., 2015a). Therefore, Pin1 overexpression may increases tendon differentiation and inhibits senescence of TSPCs.

### miRNAs Promote Tenogenic Differentiation

Aged TSPCs showed substantial upregulation of senescence marker p16^INK4A^ (Kohler et al., 2013). Osteogenic and adipogenic differentiation capacity is significantly restored by p16 ^INK4A^ knockdown in aging MSCs (Feng et al., 2014). Aged TSPCs showed decreased tenogenic differentiation capacity and upregulation of p16 which is a direct target of miR-217 (Han et al., 2017). Furthermore, TSPCs senescence may lead to defective self-renewal and altered fate (Zhou et al., 2010). TOB1 (Transducer of ERBB2, 1) is one of the TOB/B-cell translocation gene family. A study has suggested that the expression of TOB1 increased with aging during skeletal muscle development and the proliferative potential of myoblasts decreased with muscle development and aging (Yuan et al., 2011). What’s more, TOB1 is a negative regulator of BMP/Smad signaling, which negatively regulates proliferation and differentiation of osteoblasts by inhibiting the activity of receptor-regulated Smad protein (Xiong et al., 2006). A study investigated that miR-218 was found to promote the role of BMSCs in tendon-bone healing by inhibiting TOB1 in a rat supraspinatus repair model (Gao et al., 2016).

**FIGURE 1:**
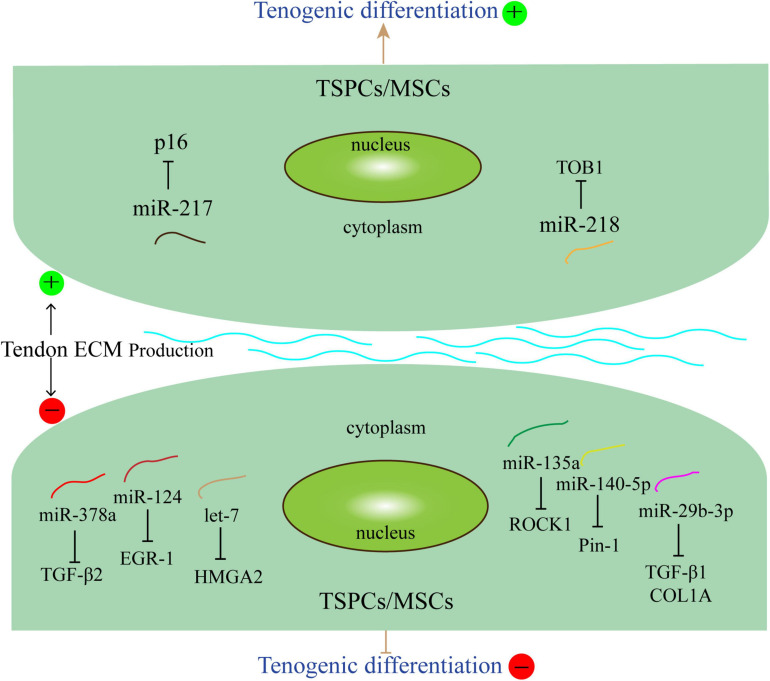
miRNAs related to tenogenic differentiation. Several miRNAs, such as miR-140-5p, miR-29b-3p, miR-378a, miR124, let-7, and miR-135a could inhibit tenogenic differentiation. Other miRNAs, including miR-217 and miR-218 could promote tenogenic differentiation. The arrows and T-shaped lines point toward mechanisms that represent promotion and inhibition, respectively.

These studies showed that miRNAs may affect the differentiation of tendon stem cells and mesenchymal stem cells through different pathways and mechanisms (Figure 1). Understanding the functional of miRNAs and their roles in physiological and pathological processes of tendons is crucial for the development of new therapies for tenogenic differentiation and repair.

## Challenges and Future Perspectives

miRNAs have been shown to regulate many potential genes related with tendon healing and tenogensis. There is great hope that tendon stem cell research could be applied to improve tendon injuries. TSPCs and MSCs are expected to be the mediums for tendon repair and regeneration, which is regulated by some director genes and a set of miRNAs coupled with some niche factors such as VEGF, TGF-β, ECM, oxidative stress, and mechanical stimuli, etc. Therefore, the study of these miRNAs may provide some potential targets for the diagnosis of tendon diseases and regeneration therapy in the future.

However, miRNAs reaching the target tendon is limited because of the poor pharmacokinetic properties of miRNAs, which means a need to produce adjuvant carrier systems that increase the stability of miRNAs. Future studies should identify how these miRNAs act on other stem cells and their extracellular microenvironments, and discover miRNAs which are responsible for tendon healing and tendon regeneration. And more importantly, future research should focus on finding the methods and approaches applicable to clinical practice.

## Author Contributions

LX and SQ conceived, designed, supervised, and commented on all the drafts of this manuscript. LD and MW contributed to the manuscript completion. All authors read and approved the final manuscript.

## Conflict of Interest

The authors declare that the research was conducted in the absence of any commercial or financial relationships that could be construed as a potential conflict of interest.

## Abbreviations

AMPK: adenosine 5′-monophosphate-activated protein kinase
BMSCs: bone marrow mesenchymal stem cells
COL 1: Type I Collagen
COL5A1: collagen type V α-1 chain
CUGBP2: CUG triplet repeat-binding protein 2
EC: endothelial cell
ECM: Extracellular Matrix
EGR-1: early growth response-1
FGF2: Fibroblast Growth Factor 2
HMGA2: high-mobility AT-hook 2
hTSPCs: human tendon-derived stem/progenitor cells
IRS1: insulin receptor substrate 1
KLF6: krüppel-like factor 6
miRBase: Microribonucleic Acid Database
miRNA/miR: microRNA
mRNA: messenger RNA
MSCs: mesenchymal stem cells
Nox4: NADPH oxidase 4
PPI: peptidyl-prolyl isomerase
pre-miRNA: miRNA precursor
RCT: rotator cuff tendon
RISC: RNA-induced silencing complex
ROCK1: Rho-associated coiled-coil protein kinase 1
Scx: scleraxis
Smad3: SMAD family member 3
TDSCs: tendon-derived stem cells
TGF-β: transforming growth factor-β
TNC: tenascin-C
TNMD: tenomodulin
TOB1: transducer of ERBB2 1
TREM-1: triggering receptor expressed on myeloid cells-1
TSCs: tendon stem cells
Tsp-4: thrombospondin-4
TSPCs: tendon stem/progenitor cells
VEGFA: vascular endothelial growth factor-A
UTR: untranslated region.
